# Comparative analysis of bacterial community structure in the rhizosphere of maize by high-throughput pyrosequencing

**DOI:** 10.1371/journal.pone.0178425

**Published:** 2017-05-25

**Authors:** Yi Yang, Na Wang, Xinyan Guo, Yi Zhang, Boping Ye

**Affiliations:** 1 School of Life Science and Technology, China Pharmaceutical University, Nanjing, PR China; 2 Nanjing Institute of Environmental Science, Ministry of Environmental Protection, Nanjing, China; Nankai University, CHINA

## Abstract

In this study, we designed a microcosm experiment to explore the composition of the bacterial community in the rhizosphere of maize and bulk soil by sequencing the V3-V4 region of the 16S rRNA gene on the Illumina system. 978–1239 OTUs (cut off level of 3%) were found in rhizosphere and bulk soil samples. Rhizosphere shared features with the bulk soil, such as predominance of *Acidobacteria*, *Proteobacteria*, *Actinobacteria*, *Bacteroidetes*, *Chloroflexi*, *Firmicutes*, *Gemmatimonadetes* and *TM7*. At genus level, many of the dominant rhizosphere genera (*Chitinophaga*, *Nitrospira*, *Flavobacterium*, etc.) displayed different patterns of temporal changes in the rhizosphere as opposed to the bulk soil, showing rhizosphere has more impact on soil microorganisms. Besides, we found that significant growth-related dynamic changes in bacterial community structure were mainly associated with phylum *Bacteroidetes*, *Proteobacteria* and *Actinobacteria* (mainly genera *Burkholderia*, *Flavisolibacter* and *Pseudomonas*), indicating that different growth stages affected the bacterial community composition in maize soil. Furthermore, some unique genera in especial Plant-Growth Promoting Rhizobacteria (PGPR) such as *Nonomuraea*, *Thiobacillus* and *Bradyrhizobium* etc., which were beneficial for the plant growth appeared to be more abundant in the rhizosphere than bulk soil, indicating that the selectivity of root to rhizosphere microbial is an important mechanism leading to the differences in the bacteria community structure between rhizosphere and bulk soil.

## Introduction

Soil microorganisms are not only an important part of soil, but also the main driver of soil nutrient cycling [1]. Soil microorganisms play critical roles in regulating soil fertility, plant health, and the cycling of carbon, nitrogen, and other nutrients.

Plants exert selective pressure on the structural and functional diversity of microbial populations through the root exudation, and in relation to soil properties, plant species, growth stage, and many other stress factors [2]. Rhizosphere was defined as a “hot spot”, rhizosphere-associated microbes are more active in this zone and possess diverse metabolic capabilities and play a crucial role in plant health and soil fertility [3]. Some bacteria that colonize the roots are plant growth-promoting rhizobacteria (PGRP), which are more closely associated with plant growth than those in bulk soil. Among them, some soil microbial communities which inhabit in rhizosphere, may restrict or inhibit the growth of potential pathogens by producing antibiotics, antifungal chemicals and insecticides [4]. Furthermore, these microbial also have the function to solubilize phospate and nitrogen. As study has reported, the genera B*acillus*, *Pseudomonas*, *Enterobacter*, *Acinetobacter*, *Burkholderia*, *Arthrobacter* and *Paenibacillus* are the common PGPRs in the rhizosphere [5].

Nelson [6] suggested the root exudates quantity and quality-mediated changes in mineral nutrition, which is essential for rhizosphere community structure. Besides, root physiology will be influenced by plant growth stage and the quality and quantity of root exudates can also be changed; thus, these changes exert a selective pressure on root-associated bacterial at different growth stages [7]. Previously studies demonstrated that seasonal variations in the activity and relative abundance of rhizosphere microorganism communities are closely related to plant type [8–10].

It has been estimated that the microorganisms which can be cultured in laboratory conditions occupied only a small part of these populations (<1%). With the continuous advances of high-throughput pyrosequencing technologies, the research on the microbial flora in the soil has become more and more thoroughly, thus provides a possibility for comprehensive analysis of the taxonomy, phylogenetic, and functional diversity of microbial communities [5, 11–12].

Since maize is one of the most important economic crops and the microbe structure in rhizosphere plays a crucial role in plant growth, it is imperative to give a better understanding of their individual roles. Thus, in this work we have carried out a microcosm experiment, with maize as an ideal plant for investigation. The aim was to assess and add some new insight into the influence of growth stage on composition of the bacterial community in the rhizosphere and bulk soil. It was also of interest to identify dominant and sensitive soil bacteria and in the hope of contributing to provide relevant data for further research.

## Materials and methods

### Experiment design

The study was permitted and approved by the Ministry of Environmental Protection, China. The land accessed was not privately owned or protected. No protected species were sampled. Manure was collected from pigs at the experimental farm of Jiangsu Province Tomorrow Agriculture and Animal Husbandry Science and Technology Co., Ltd. The soil used for microcosm were paddy soil from the experimental farm of Nanjing Institute of Environment Science, MEP with 49.8 g·kg^-1^ organic carbon content, a pH of 6.23, an ion exchange capacity of 18.0 cmol·kg^-1^, 19.4% clay, 75.8% silt, and 4.8% sand. Manure and soil were mixed with an electric mixing machine at a ratio of 1:25 (w/dw), and the soil was filled into polystyrene tubes (inner diameter: 17cm, height: 60cm) that were planted with maize (*Zea mays L*.). The plants were kept in green house with a day/night cycle of 16:8 h. Water loss was replenished daily to keep soil moisture varied between 8.5 and 15.2% (w/dw).

### Sampling and DNA extraction

Soil samples were collected after maize had grown for 14d, 35d, and 63d. The maize roots were shaken vigorously to remove loosely adhered soil which were defined as bulk soil (BK), while the soil that tightly adhered to the roots was regarded as rhizosphere (RH), soil samples were named BK-1, BK-2, BK-3, and RH-1, RH-2, RH-3, respectively. All samples transported on ice to be stored at 4°C before analysis in the lab. The total DNA extraction from rhizosphere and bulk soil samples was performed employing a PowerSoil DNA Isolation Kit (USA, MOBIO) according to the protocol. The concentration and quality of the extracted total DNA were examined by UV spectrophotometry (Nanodrop 1000, Thermo, USA) and 1% agarose gel electrophoresis.

### High-throughput 16s rRNA pyrosequencing

For gene libraries construction, the bacterial 16S rRNA gene was amplified by PCR with a set of primers targeting the hypervariable V3–V4 region (about 460 bp). The V3 forward primer was 5′–ACTCCTACGGGAGGCAGCAG-3′ and V4 reverse primer was 5′- TACNVGGGTATCTAATCC-3′. The PCR procedures were as follows: 94°C for 3min as an initial denaturation, 30 cycles of 94°C for 30s, 62°C for 30s, 70°C for 45s, and 72°C for 7 min as a final extension. A total volume of reaction mixture was 50μL which consisted of 1×Pfx Amplification Buffer (Invitrogen, USA), 0.4 mM dNTP, 2 mM MgSO4, 0.4 μM each primers, 1 μL of template DNA and 2 U of Platinum^®^ Pfx DNA Polymerase (Invitrogen, USA). The extracted DNA was subject to high-throughput pyrosequencing using TruSeq SBS Kit V3 on Illumina Hiseq 2000 (Illumina, USA). The Illumina pyrosequencing strategy was “Index 101 PE” (Paired End sequencing, 101-bp reads and 8-bp index sequence), which generates nearly equal amount of clean reads for each sample. For quality control, the raw reads containing three or more “N” or contaminated by adapter (>15 bp overlap) were removed.

### Analysis of microbial community and biodiversity

After 454 pyrosequencing, the clean high-quality sequence were grouped into operational taxonomic units (OTUs) using the Quantitative Insights into Microbial Ecology (QIIME) at 97% sequence identity [13]. Representative sequences from each OTU were assigned down to the phylum and genus using software RDP MultiClassifier at 80% confidence [14]. Richness and diversity indices including operational taxonomic units (OTUs), Chao estimator and abundance-based coverage estimator (ACE), as well as rarefaction curves, were calculated using Mothur.

## Results

### Overall structural variance of bacterial communities

After sequencing the V3-V4 region, a total of 218905 raw reads were generated from soil samples. As shown in Table 1, after filtering the low quality reads using the QIIME' standard pipeline and trimming the primers, adapters and barcodes, a total of 35603–36388 and 35587–36492 high quality clean reads were obtained for rhizosphere and bulk soil, respectively.

**Table 1:** Comparison of the estimated OTU richness and diversity indexes of the 16S rRNA gene libraries for clustering at 97% identity.

Sample^a^	Shannon^b^	Raw reads	Clean reads	Chao	ACE^c^	Simpson	OTUs^d^
RH1	5.330	36076	35603	1276.304	1285.612	0.015	1009
BK1	5.240	35962	35587	1335.727	1337.056	0.019	978
RH2	6.228	36556	36016	1490.337	1488.857	0.004	1239
BK2	5.769	36350	35880	1443.947	1428.056	0.009	1160
RH3	5.378	36956	36388	1479.634	1479.729	0.022	1167
BK3	5.495	37005	36492	1322.793	1335.411	0.015	1103

^a^ Sample names 1, 2 and 3 indicated samples collected after maize had grown for 14d, 35d, and 63d, respectively. RH and BK represent rhizosphere and bulk soil.

^b^ Shannon diversity index calculated using Mothur.

^c^ Abundance-based coverage estimator calculated by Mothur.

^d^ Number of operational taxonomic unit calculated with Mothur.

For detail, 978–1239 OTUs (cut off level of 3%) were found in rhizosphere and bulk soil samples. The OTUs first increased and then decreased in all soil samples as maize growth progressed, and were consistently higher in rhizosphere than those in bulk soil.

The Shannon index yielded values ranging from 5.330 to 6.228 for rhizosphere and from 5.240 to 5.769 for bulk soil, suggesting a relatively higher diversity of bacterial sequences in the rhizosphere. The values of ACE for samples from rhizosphere were ranged from 1285.612 to 1479.729, whereas those for samples from bulk soil were 1337.056 to 1335.411. Overall, both the index of Chao, ACE, Shannon and Simpson revealed similar trends, with higher values observed for rhizosphere samples, when compared with samples from bulk soil, indicating that the higher abundance of bacterial community in rhizosphere, however, the difference among them was not significant.

**Fig 1:**
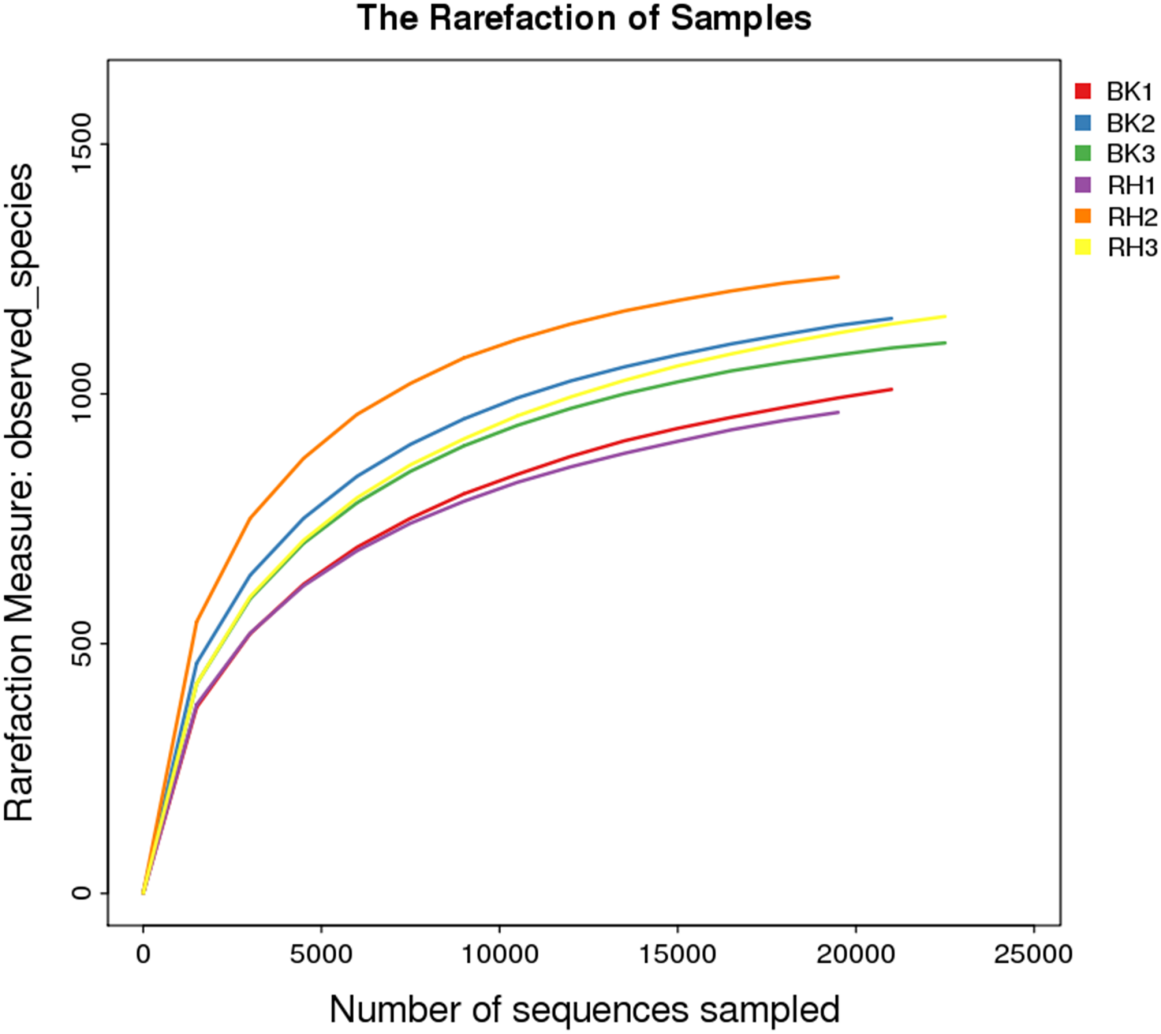
Rarefaction curves based on observed species value.

Rarefaction curve was the common used method to compare species diversity in the ecosystem. The rarefaction curves of the two soil groups were also drawn in this study (Fig 1). Results found that the rarefaction curves of these samples didn’t approach the asymptote, which indicated that each sample showed highly diversified microbial communities and further sequencing would have generate more OTUs in each sample. In this study, these curves indicated that OTUs were higher in rhizosphere than in bulk soil at each sampling time, with the OTUs increased in 35d and then decreased in 63d.

### Differences in bacterial community composition

**Fig 2:**
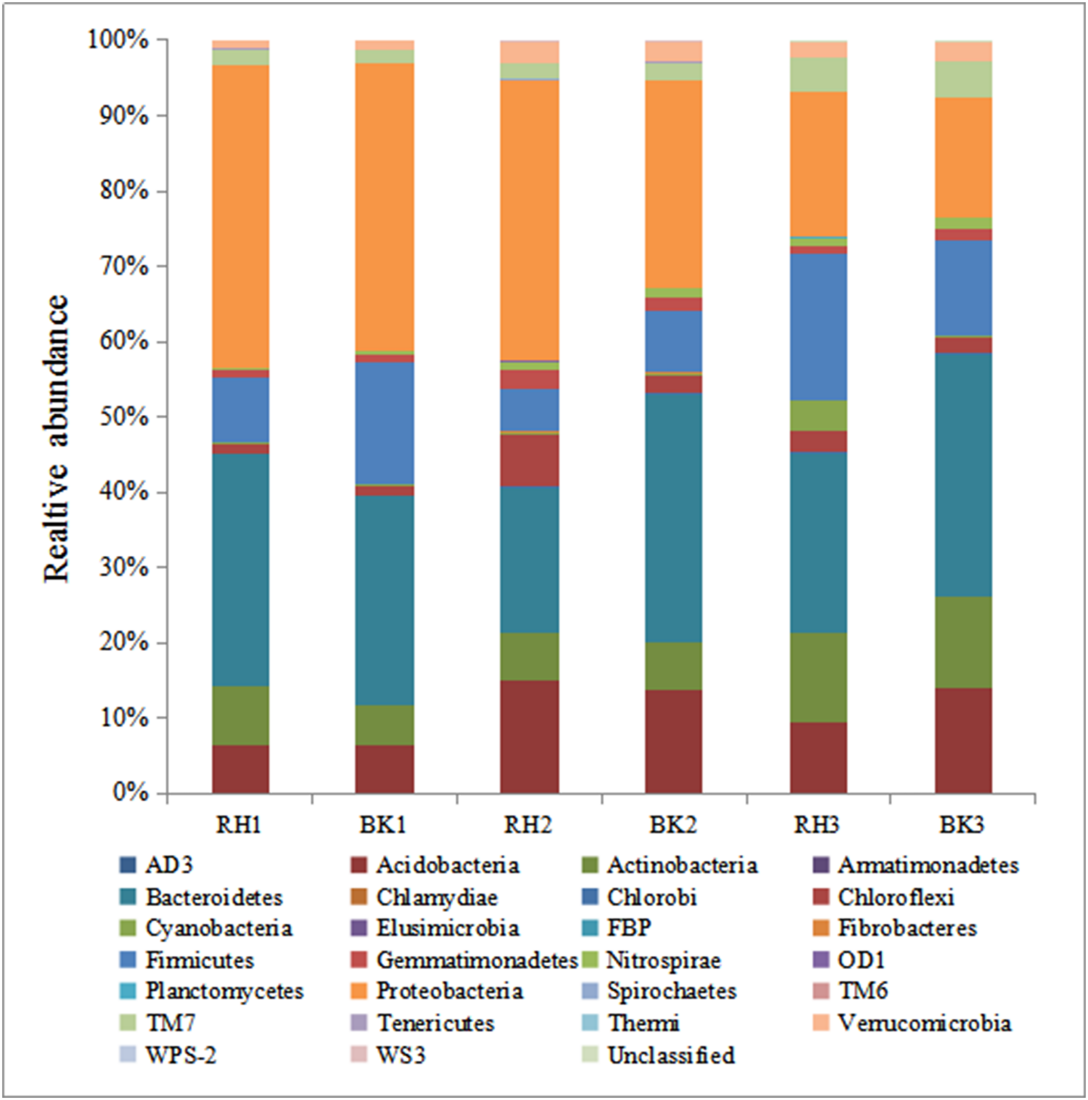
Relative abundance of the rhizosphere and bulk soil samples at phylum level.

The average relative abundances are presented in Fig 2, and the classified sequences were associated with 28 phyla, but only 9 were found at a relative abundance of >1%. The major phylum groups were *Acidobacteria*, *Proteobacteria*, *Actinobacteria*, *Bacteroidetes*, *Chloroflexi*, *Firmicutes*, *Gemmatimonadetes* and *TM7*, accounted for more than 90% of bacterial sequences in both rhizosphere and bulk soil.

Fig 2 exhibits the main phylum compositions of rhizosphere and bulk soil samples. Rhizosphere was mainly composed of *Proteobacteria* (19.3%~40.2%), *Bacteroidetes* (19.1%~30.8%), *Firmicutes* (5.4%~19.4%), *Actinobacteria* (6.3%~11.7%) and *Acidobacteria* (6.5%~15.0%). Bulk soil was mostly comprised by *Proteobacteria* (15.8%~38.3%), *Bacteroidetes* (27.2%~32.2%), *Firmicutes* (8.1%~16.2%), *Acidobacteria* (6.3%~14.1%) and *Actinobacteria* (5.4%~11.9%). *WS3*, *Chlorobi*, *Cyanobacteria*, *Elusimicrobia*, *Nitrospirae*, *Planctomycetes* and *Tenericutes* were present in most soil samples, but at relatively low abundance (Fig 2).

Many sequences could not be classified but were abundant in rhizosphere and bulk soil (1.22%~18.95%), demonstrating that soil remains a challenging reservoir of biodiversity. Moreover, the ratio of sequences in rhizosphere and bulk soil was 63d>35d>14d, indicating that bacterial diversity was modified with root activity at different sampling times.

A thorough investigation at the genus level showed an enrichment trend of predominant bacterial groups in soil samples. The relative abundance of any genus was <7% in each sample, implying high bacterial diversity in the two sample groups. As shown in Tables 2 and 3, 44 genera were obtained in all soil samples. *Chitinophaga*, *Flavisolibacter*, *Nitrospira*, *Pseudomonas* and *Streptomyces* which were predominantly found in the rhizosphere and bulk soil, belong to *Bacteroidetes*, *Proteobacteria* and *Actinobacteria*, respectively.

**Table 2:** Distribution and changes of different genera in maize rhizosphere and bulk soil of different sampling times.

Genera	Relative abundance (%)						Increasing of relative abundance (%)			
	RH1	RH2	RH3	BK1	BK2	BK3	RH2	RH3	BK2	BK3
*Acinetobacter*	0.90	0.12	0.62	0.89	0.39	0.01	-86.78	423.43	-56.14	-97.74
*Actinomadura*	0.08	0.02	0.95	0.08	0.04	1.50	-69.38	3719.66	-46.07	3549.86
*Aeromicrobium*	0.15	0.56	0.21	0.08	0.24	0.25	279.53	-63.19	198.91	4.52
*Agrobacterium*	0.37	0.76	0.75	0.50	0.73	0.26	108.24	-1.89	45.62	-64.99
*Amycolatopsis*	0.19	0.01	0.57	0.11	0.09	0.44	-91.99	3747.85	-20.10	381.07
*Arthrobacter*	0.65	0.41	0.18	0.75	0.56	0.13	-36.92	-57.05	-24.28	-77.27
*Burkholderia*	1.24	0.33	0.67	0.60	0.31	0.36	-73.57	104.48	-47.91	14.09
*Candidatus_Koribacter*	0.51	0.45	0.56	0.58	0.52	0.83	-12.27	24.06	-9.63	58.03
*Candidatus_Solibacter*	0.59	1.57	0.55	0.61	0.85	1.04	166.18	-64.92	38.98	21.61
*Caulobacter*	0.62	0.32	0.18	0.37	0.23	0.18	-49.13	-44.30	-38.54	-22.25
*Chitinophaga*	6.95	1.54	3.78	5.18	6.24	6.49	-77.79	145.09	20.50	4.00
*Clostridium*	0.57	0.45	1.57	1.19	0.51	0.89	-20.38	247.92	-57.22	75.28
*Pseudomonas*	4.01	1.02	0.01	3.67	0.43	0.01	-74.50	-98.76	-88.22	-97.69
*Cupriavidus*	0.36	0.32	0.07	0.35	0.51	0.03	-9.76	-77.80	45.78	-94.75
*DA101*	0.12	0.41	0.45	0.28	0.82	0.69	228.38	10.76	197.54	-15.23
*Devosia*	0.20	0.72	0.41	0.19	0.52	0.14	261.94	-43.61	182.71	-73.80
*Dokdonella*	0.07	0.53	0.04	0.03	0.49	0.11	695.77	-92.07	1609.77	-77.29
*Dyadobacter*	0.71	0.80	1.49	1.11	1.88	0.77	13.97	84.95	69.94	-58.82
*Flavisolibacter*	1.60	1.24	2.09	1.25	2.28	2.64	-22.61	69.08	82.64	15.81
*Flavobacterium*	1.76	0.95	0.98	2.07	1.21	0.87	-46.25	3.54	-41.37	-28.39
*Glycomyces*	0.08	0.00	0.74	0.01	0.01	0.37	-93.88	14839.71	43.81	2621.27
*Lysobacter*	0.58	0.27	0.27	0.52	0.70	0.26	-53.06	-1.23	35.46	-62.77

**Table 3:** Distribution and changes of different genera in maize rhizosphere and bulk soil of different sampling times.

Genera	Relative abundance (%)						Increasing of relative abundance (%)			
	RH1	RH2	RH3	BK1	BK2	BK3	RH2	RH3	BK2	BK3
*Mesorhizobium*	0.20	0.46	0.72	0.15	0.23	0.40	122.77	57.77	49.81	74.93
*Methylibium*	0.96	0.58	0.13	1.25	0.83	0.18	-39.39	-76.78	-33.54	-78.76
*Nannocystis*	0.70	0.20	0.07	0.77	0.26	0.05	-71.47	-66.05	-66.27	-79.54
*Nitrospira*	4.83	0.87	2.59	4.07	4.15	5.56	-81.91	196.12	2.04	33.99
*Niastella*	0.31	1.08	0.83	0.36	1.00	1.34	253.03	-22.55	176.27	34.02
*Nocardia*	0.27	0.02	0.39	0.19	0.16	0.58	-90.70	1478.79	-16.11	263.75
*Pedobacter*	0.43	0.35	0.89	0.65	0.69	0.56	-19.02	157.07	5.67	-18.90
*Comamonas*	0.68	0.58	0.03	1.55	0.19	0.04	-14.21	-94.20	-87.94	-81.04
*Pseudoxanthomonas*	0.27	2.59	0.07	0.58	0.54	0.15	851.69	-97.23	-7.27	-72.00
*Ralstonia*	0.64	0.04	0.13	0.56	0.01	0.02	-93.83	218.30	-97.54	61.98
*Rhodoplanes*	0.28	0.78	0.74	0.23	0.32	0.71	177.06	-4.85	41.81	119.01
*Roseateles*	0.52	0.08	0.08	0.43	0.25	0.02	-84.72	-4.51	-42.05	-91.16
*SMB53*	1.07	0.89	4.17	2.60	1.53	2.08	-17.17	369.92	-41.11	35.94
*Sorangium*	0.60	0.58	0.03	2.52	1.37	0.07	-3.38	-95.61	-45.83	-95.14
*Sphingobacterium*	1.76	0.11	0.46	1.35	0.52	0.12	-93.81	320.54	-61.31	-76.34
*Sphingomonas*	0.30	0.59	0.98	0.37	0.52	0.61	96.67	66.19	41.94	16.79
	0.78	0.17	0.03	1.23	0.38	0.01	-78.41	-80.03	-69.28	-97.66
*Steroidobacter*	0.29	0.44	0.45	0.36	0.64	0.49	52.71	3.21	80.24	-24.18
*Streptomyces*	4.11	1.49	4.45	2.30	2.15	5.25	-63.68	197.79	-6.69	144.20
*Thermomonas*	0.36	0.59	0.39	0.44	0.51	0.33	61.66	-33.10	17.76	-35.50
*Turicibacter*	0.24	0.16	0.67	0.39	0.25	0.30	-32.63	308.97	-35.69	18.39
*Variovorax*	0.75	0.36	0.37	0.78	0.70	0.24	-51.59	3.48	-9.97	-65.29

The relative abundance of *Streptomyces*, *Burkholderia*, *Caulobacter*, *Devosia*, *Glycomyces* and *Ralstonia* was higher in rhizosphere than bulk soil, while *DA101*, *Dyadobacter*, *Niastella*, *Pseudomonas* and *Sorangium* demonstrated the reverse.

The OTU abundance varied from sampling times. For example, *Vrhodoplanes*, *Streptomyces*, *Nocardia*, *Actinomadura*, *Amycolatopsis*, *Dokdonella* and *Niastella* significantly increased in 35d and/or 63d in bulk soil, rising by 119.01%-3549.86%. In the *rhizosphere*, *Nocardia*, *Pseudoxanthomonas*, *SMB53*, *Sphingobacterium*, *Turicibacter*, *Actinomadura*, *Amycolatopsis* and *Dokdonella* was elevated by 308.97%-3747.85%, compared to the last sampling time.

The abundance of *Niastella* and *Flavisolibacter* in rhizosphere was decreased in 35d and then increased in 63d, while difference occurred in bulk soil, with a continued increase from 14d to 63d. The results suggest that bacterial community composition was altered markedly to a greater extent in the rhizosphere than bulk soil, indicating that rhizosphere bacteria may respond first to environmental changes ahead of bulk soil.

### Comparison on microbial genera

In this study, some unique genera in rhizosphere and bulk soil were found, respectively (Table 4). Among them, 8 special genera were obtained in rhizosphere, according to the function can be divided into: anti-bacteria (*Nonomuraea*), nitrogen fixing bacteria (*Phenylobacterium*, *Inquilinus*, *Bradyrhizobium* and *Nitrosovibrio*), phosphate solubilizing bacteria (*Thiobacillus* and *Erwinia*), and pathogenic bacteria (*Rickettsia*); while, 5 unique genera detected in bulk soil were divided into: phosphate solubilizing bacteria (*Gemmatimonas*), nitrogen fixing bacteria (*Azospirillum* and *Actinomycetospora*), pathogenic bacteria (*Aquicella* and *Conexibacter*). In rhizosphere, most unique genera bacteria belongs to PGPR, and the main function were phosphorus solubilizing, nitrogen fixation, and degrade cellulose, while in bulk soil belongs to PGPR and pathogenic bacteria.

**Table 4:** Comparison on microbial genera between rhizosphere and bulk soil of maize.

Genus	Function*	Rhizosphere	Bulk soil
*Nonomuraea*	Produce antibiotics	+	—
*Phenylobacterium*	Nitrogen fixation	+	—
*Thiobacillus*	Phosphorus solubilizing	+	—
*Erwinia*	Phosphorus solubilizing	+	—
*Inquilinus*	Nitrogen fixation	+	—
*Nitrosovibrio*	Nitrogen fixation	+	—
*Bradyrhizobium*	Symbiotic nitrogen fixation	+	—
*Rickettsia*	Pathogenic bacteria	+	—
*Azospirillum*	Phosphorus solubilizing	—	+
*Gemmatimonas*	Nitrogen fixation	—	+
*Actinomycetospora*	Associative nitrogen fixation	—	+
*Aquicella*	Pathogenic bacteria	—	+
*Conexibacter*	Pathogenic bacteria	—	+

+: presence of the genus in the environment;

—: absence of the genus in the environment.

*: the function description of each genus is based on *Taxonomic Outline of the Prokaryotes*, Bergey’s Manual of Systematic Bacteriology (Eighth edition)

## Discussion

Soil is a very complex ecological system, and the soil microorganism plays an important role in the soil. High-throughput pyrosequencing is considered to be a good method to study soil microbial diversity, which can be used to carry out high quality sequencing of DNA specific sections of soil microbial communities. In this study, the diversity, composition, and relative abundance of bacterial community in rhizosphere and bulk soil were represented at different sampling times during the maize growth process, via Illumina high-throughput sequencing. The results provided insights into the effects of alterations in maize root exudates on microbial community.

After sequencing the V3-V4 region of 16S rRNA, 215903 effective sequences were obtained in total with 35983 sequences per sample on average, then clustered into 978~1167 OTUs at 97% similarity level. By comparing with the database, we found that the composition of microbial community in rhizosphere was similar but higher than bulk soil on the phylum level. However, with the growth period of maize, the relative abundance of each phylum varying in soil samples, which may be related to soil structure, nutrients, root exudates and other factors.

*Proteobacteria* is the predominant phylum in rhizosphere may due to their rapid growth rates and, this nutrient-rich environment was suit for the phylum Proteobacteria, or certain classes within this phylum [15]. This genus bacterium is mostly Gram-negative and many are responsible for nitrogen fixation and polycyclic aromatic hydrocarbons. In according with previous study that *Acidobacteria* were similar regardless of crop variety (grass or wheat) and land management practice [4]. The number of *Bacteroidetes* in intestinal accounted for more than half of total bacteria, thus can be widely detected in manure and manure-amended soil. *Actinobacteria* are widely founded in soil and water ecosystem and play a critical role in the decomposition and humus formation process [16]. Overall, these findings were in consistent with previous studies on bacterial communities in soil [17, 18]. These authors concluded that major soil phyla were comprised of *Proteobacteria*, *Actinobacteria*, *Acidobacteria*, *Bacteroidetes*, *Firmicutes* and *Plantcomycetes*, although recognizing that their relative abundances vary with the study site.

There were several key microbial groups that displayed different temporal dynamics in rhizosphere from that in the bulk soil. In fact, many of the dominant rhizosphere genera (*Chitinophaga*, *Nitrospira*, *Flavobacterium*, etc.) displayed different patterns of temporal changes in the rhizosphere as opposed to the bulk soil (Tables 2 and 3), showing rhizosphere has more impact on soil microorganisms. It can be expected that the composition and chemical nature of root exudates will change over the plant growth cycle. In this study, we found that significant growth-related dynamic changes in bacterial community structure were mainly associated with phylum *Bacteroidetes*, *Proteobacteria* and *Actinobacteria* (mainly genera *Burkholderia*, *Flavisolibacter* and *Pseudomonas*). Members of *Burkholderia* were enriched in the rhizosphere, possibly due to their versatile abilities to utilize root metabolites, degrade aromatic compounds [19] and produce anti-microbial substances. *Pseudomonas* has rapid growth and is therefore good colonizers in soil. *Pseudomonas* can use various substrates as nutrients and survive under different stressing conditions. Also its ability to produce various compounds, such as antibiotics, polysaccharides and siderophores are crucial to its success. Li et al [20] reported that the genera *Flavobacterium* may be copiotrophic and fast-growth bacteria, able to exploit a transient niches for nutrition and others at the early growth stage of maize. The decline of *Flavobacterium* at later stages may be explained by the reduction of quantity and quality of organic carbon, or interspecies competition for resources.

There were more than 2.6×10^29^ live cells in soil [17], but majority of them can be found in rhizosphere. The roots of plant seem to having a stronger impact on microbial activity. Some microorganisms and some especial bacteria, which are known as Plant-Growth Promoting Rhizobacteria (PGPR) benefit plant growth and health. These bacteria can increase the availability of nutrients for the plant in the rhizosphere. Previous researches had also shown that fertilizer amount can be reduced after adding the PGPR bacteria into crops [21].

It is essential to give insight to the microbial biodiversity since the bacterial diversity and soil function are correlated. Most of the bacteria in the rhizosphere are phosphate solubilizing, nitrogen, fixing and plant disease suppressing which helps to improve the crop yield and protection. In rhizosphere, most unique genera bacteria belongs to PGPR, and the main function were phosphorus solubilizing, nitrogen fixation, and degrade cellulose, while in bulk soil belongs to PGPR and pathogenic bacteria.

These changes in the bacterial species may have a close relationship with the root exudates of maize. For example, Nitrogen fixing bacteria are very sensitive to sugar and amino acids in root exudates, thus leading to a large number of bacteria with nitrogen fixing function in the rhizosphere [22]. Besides, pH, soil structure, organic matter and nutrient levels in the rhizosphere and bulk soil were different and thus can led to more beneficial microbes dwelling in the rhizosphere region [23]. Meanwhile, rhizosphere can alter plant physiological and biochemical processes by changing the rhizosphere nutrition status and effecting plant hormone content, which makes the interaction between rhizosphere and microbes develop toward to the more conducive direction of both sides.

## Conclusion

At this present study, we have analyzed the maize bacterial community in the rhizosphere and bulk soil at different growth stages. Overall, the abundance and diversity of microbial community in rhizosphere were higher than bulk soil, and related closely with maize growth stages. Besides, some growth-promoting microorganisms beneficial for the plant growth appeared to have been more abundant in the rhizosphere than bulk soil, indicating that selectivity of root to rhizosphere microbial is an important mechanism leading to the differences in the bacteria community structure between rhizosphere and bulk soil.
